# Enhanced Cellular Uptake and Pharmacokinetic Characteristics of Doxorubicin-Valine Amide Prodrug

**DOI:** 10.3390/molecules21101272

**Published:** 2016-09-22

**Authors:** Yohan Park, Ju-Hwan Park, Suryeon Park, Song Yi Lee, Kwan Hyung Cho, Dae-Duk Kim, Won-Sik Shim, In-Soo Yoon, Hyun-Jong Cho, Han-Joo Maeng

**Affiliations:** 1 College of Pharmacy, Inje University, Gyeongnam 50834, Korea; yohanpark@inje.ac.kr (Y.P.); mbong16@gmail.com (S.P.); chokh@inje.ac.kr (K.H.C.); 2 College of Pharmacy and Research Institute of Pharmaceutical Sciences, Seoul National University, Seoul 08826, Korea; cyclone5pjh@naver.com (J.-H.P.); ddkim@snu.ac.kr (D.-D.K.); 3 College of Pharmacy, Kangwon National University, Gangwon 24341, Korea; heymush@kangwon.ac.kr; 4 College of Pharmacy, Gachon University, Incheon 21936, Korea; wsshim@gachon.ac.kr; 5 College of Pharmacy and Natural Medicine Research Institute, Mokpo National University, Jeonnam 58554, Korea; yooninsu99@naver.com

**Keywords:** amino acid transporters, cancer cell, cellular uptake, doxorubicin, valine prodrug, amide bond

## Abstract

In this study, we synthesized the valine (Val)-conjugated amide prodrug of doxorubicin (DOX) by the formation of amide bonds between DOX and Val. The synthesis of the DOX-Val prodrug was identified by a proton nuclear magnetic resonance (^1^H-NMR) assay. In the MCF-7 cells (human breast adenocarcinoma cell; amino acid transporter–positive cell), the cellular accumulation efficiency of DOX-Val was higher than that of DOX according to the flow cytometry analysis data. Using confocal laser scanning microscopy (CLSM) imaging, it was confirmed that DOX-Val as well as DOX was mainly distributed in the nucleus of cancer cells. DOX-Val was intravenously administered to rats at a dose of 4 mg/kg, and the plasma concentrations of DOX-Val (prodrug) and DOX (formed metabolite) were quantitatively determined. Based on the systemic exposure (represented as area under the curve (AUC) values) of DOX-Val (prodrug) and DOX (formed metabolite), approximately half of DOX-Val seemed to be metabolized into DOX. However, it is expected that the remaining DOX-Val may exert improved cellular uptake efficiency in cancer cells after its delivery to the cancer region.

## 1. Introduction

To date, many various approaches have been introduced to increase efficacies and reduce unwanted effects in chemotherapy. Diverse formulations, especially nano-sized carriers, have been developed to selectively deliver anticancer drugs to the tumor region [1,2,3]. However, the translation from preclinical to clinical application was not easy due to the toxicity of formulation additives and other components. The prodrug strategy has gained much attention due to its ability to avoid the typical disadvantages (i.e., side effects) of pharmaceutical excipients for clinical application. Prodrugs are derivatives of drug molecules undergoing chemical and/or enzymatic transformation in the body [4]. It is known that approximately 5%–7% of drugs can be classified as a prodrug. Prodrug approaches have been used to improve physicochemical and biopharmaceutical properties of pharmacologically active agents [4]. Aqueous solubility and pharmacokinetic properties (i.e., absorption, distribution, metabolism, and excretion) can be improved and altered by prodrug synthesis [5,6]. Various substances (i.e., natural and synthetic polymers, lipids, nucleic acids, proteins, peptides, and small chemicals) can be linked to the drug molecule to modify its chemical structure [7,8,9,10,11,12].

For chemotherapy, a variety of prodrugs of anticancer agents have been developed [13,14]. The aims of a prodrug approach in cancer therapy include the following properties: prolonged circulation in the blood stream, selective cancer cell uptake, and overcoming the multidrug resistance (MDR) phenomenon in cancer cells [15,16,17]. Numerous moieties have been introduced to synthesize prodrugs in anticancer agents [13,15].

In particular, amino acid transporters, such as l-type amino acid transporter (LAT1)-mediated cellular uptake of anticancer drugs, can be used as a versatile implementation for chemotherapy [18,19]. In our previous reports [20,21], we have conducted experiments in which the amino acids (i.e., tyrosine, valine) were introduced to two anticancer agents (e.g., lapatinib, SN-38) to improve their cellular uptake or anticancer activities via amino acid transporters. The two prodrug strategies for anticancer agents with an amino acid moiety clearly demonstrated that the amino acid transporters, including amino acid transporter B°^,+^ (ATB°^,+^), sodium-dependent amino acid transporters (i.e., ATA1, ATA2, and ASCT2), sodium-independent amino acid transporter, and LAT1, are involved in the transport of the amino acid prodrug of anticancer agents [20,21]. However, these two studies presented some critical limitations. Namely, the ester valine prodrug of SN-38 (Val-SN-38) was metabolically unstable since the ester linkage is susceptible to being broken by abundant esterase in vivo [20]. Although Maeng et al. [21] first introduced the amide linkage to lapatinib—a novel tyrosine kinase inhibitor with improved metabolic stability for overcoming the MDR phenomenon in cancer cells—studies for the anticancer prodrug with amino acid via the amide linkage regarding the direct uptake of the amino acid prodrug or in vivo pharmacokinetics have not yet been extensively investigated.

Herein, the Val-modified amide prodrug of doxorubicin (DOX-Val) was successfully synthesized and characterized with respect to the cellular accumulation efficiency and cellular distribution of DOX-Val in vitro. Its improved cellular uptake efficiency in MCF-7 cells was demonstrated by flow cytometry and confocal laser scanning microscopy (CLSM). The pharmacokinetics of the prodrug (DOX-Val) and its metabolite (DOX) after intravenous injection in rats was also investigated.

## 2. Results and Discussion

### 2.1. Synthesis and Characterization of DOX-Val

**Figure 1:**
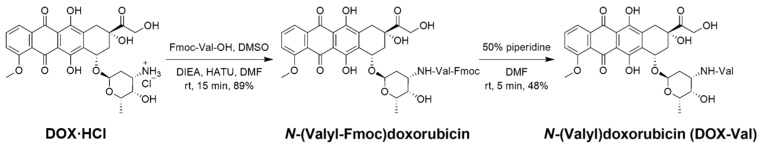
Synthetic scheme of *N*-(valyl)doxorubicin (DOX-Val) from doxorubicin hydrochloride (DOX·HCl).

According to the previous report [22], *N*-(valyl)doxorubicin (DOX-Val, 3) was synthesized from doxorubicin hydrochloride (DOX·HCl) (1) in two steps (Figure 1). The amide-coupling reaction of DOX (1) and *N*-(9-fluorenylmethoxycarbonyl)-l-valine (Fmoc-Val-OH) afforded *N*-(valyl-Fmoc)doxorubicin (2) (89% yield). DOX-Val (3) was obtained by the deprotection of the Fmoc group with a 50% piperidine condition (48% yield). Through the protection and deprotection of the amine group in the Val residue, selective amide bond formation between the carboxylic acid group of Val and the amine group of DOX occurred. In previous studies [20,21], the Val residue was conjugated to anticancer agents via an ester bond or amide bond. Due to the presence of various endogenous esterases in biological fluids, the Val-conjugated prodrug linked via an ester bond was prone to degradation [23,24]. In this investigation, Val was linked via an amide bond to DOX to improve its stability in biological fluids [21]. The successful synthesis of DOX-Val was verified by ^1^H-NMR, and the purity of DOX-Val was examined by high performance liquid chromatography (HPLC).

### 2.2. Cellular Uptake of DOX-Val

MCF-7 (amino acid transporters, ATA1, ATA2, ASCT2, LAT-1, and MDR1-positive; human breast adenocarcinoma cell) has been used in this study to compare the cellular accumulation efficiency between DOX and DOX-Val. Our previous study clearly demonstrated that the expression level of amino acid transporters—ATA1, ATA2, ASCT2, and LAT-1—existed in MCF-7 cells by semi-quantitative polymerase chain reaction (PCR) [20].

**Figure 2:**
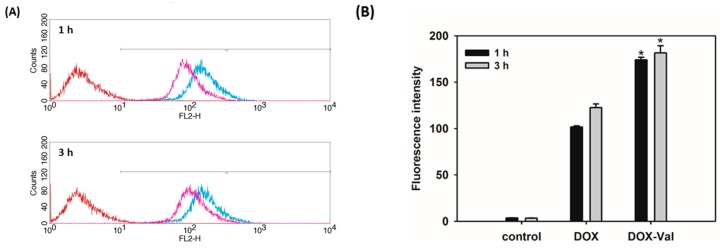
Cellular accumulation efficiency of DOX-Val evaluated by flow cytometry in MCF-7 cells. DOX and DOX-Val (10 μM) were incubated for 1 h and 3 h in MCF-7 cells. (**A**) Histograms of all experimental groups, control (red), DOX (pink), and DOX-Val (blue), are shown; (**B**) The mean value of the fluorescence intensity of each group is presented. Data are presented as means ± standard deviation (*n* = 3). * *p* < 0.05, compared with DOX group.

**Figure 3:**
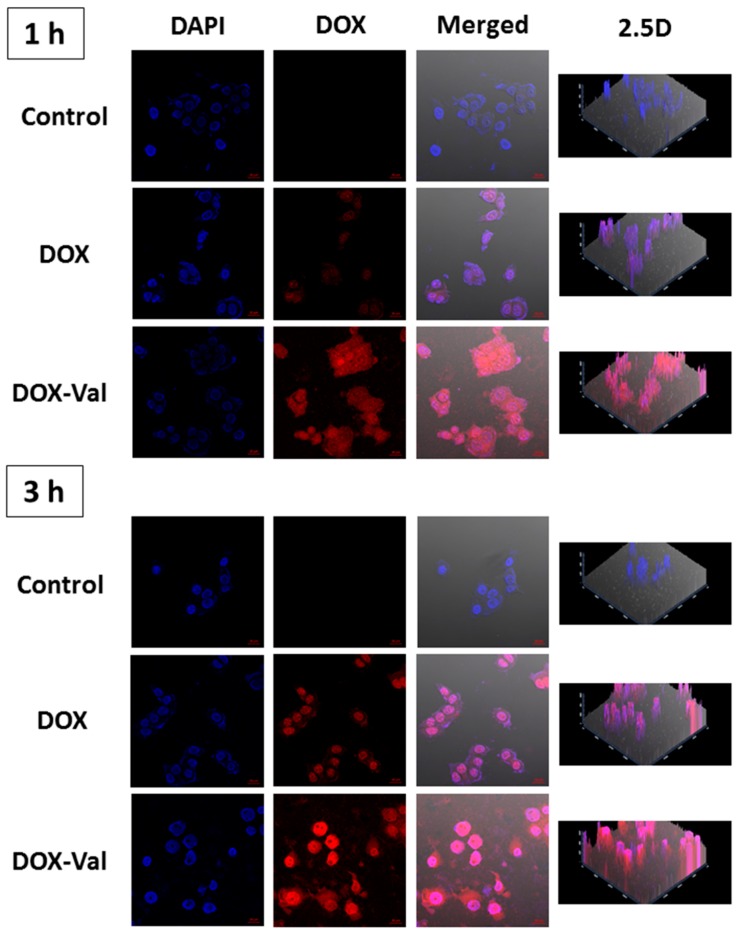
Cellular distribution of DOX and DOX-Val in MCF-7 cells observed by CLSM. DOX and DOX-Val (10 μM) were incubated for 1 h and 3 h in MCF-7 cells. Red and blue colors indicate DOX and 4′,6-diamidino-2-phenylindole (DAPI), respectively. DAPI, DOX, merged, and 2.5 D images are presented. The 2.5 D image can provide spatial information between 2D and 3D images. The length of the scale bar in the image is 20 μm.

The cellular accumulation efficiency and cellular distribution of DOX-Val were studied by flow cytometry and CLSM (Figure 2 and Figure 3). To monitor the participation of Val in the cellular uptake, the cell culture medium without glutamine was used in cellular uptake studies. Although the affinity of Val to LAT-1 was different in accordance with the cell culture systems [25,26], the improved cellular uptake of the Val-conjugated prodrug through amino acid transporters was already demonstrated in our previous reports [20,21]. Namely, the addition of Val to lapatinib exerted a significant inhibition of glutamine uptake in the various cancer cells such as MDA-MB-231, MCF7, and A549 cancer cells, but not in the normal kidney cells, the MDCK-II cell line [21], likely suggesting that the enhanced uptake of the Val-added anticancer agents is unlikely to occur in normal organ cells rather than cancer cells, which highly express various amino acid transporters. Moreover, it was demonstrated that the addition of Val to SN-38 did not increase the apparent permeability in the parallel artificial membrane permeability assay (PAMPA) [20], indicating that increased cellular accumulation by the Val addition is not due to the increased membrane permeability via passive diffusion. As shown in Figure 2, the cellular accumulation efficiency of DOX and DOX-Val in MCF-7 cells was measured after 1 h and 3 h incubation. Consistent with our previous studies [20,21], DOX-Val exhibited a 71% and 48% stronger fluorescence intensity compared with the DOX group after 1 h and 3 h incubation, respectively (*p* < 0.05). The cellular distribution of DOX and DOX-Val was also measured by CLSM after 1 h and 3 h incubation (Figure 3). DOX-Val exhibited a stronger fluorescence signal compared with the DOX group for both 1 h and 3 h incubation. DOX and DOX-Val seem to be distributed mainly in the nucleus of cells, as shown in Figure 3. Although the intervention of specific types of amino acid transporters should be further elucidated, the augmented cellular uptake efficiency of the Val-modified prodrug (DOX-Val) was accomplished in the glutamine-free cell culture media in this study. The improved cellular accumulation of DOX-Val, as compared with DOX, may lead to enhanced cytotoxicity against cancer cells. In this study, a direct study to confirm the possibility to overcome the MDR phenomenon using DOX-resistant cancer cells was not studied. Further studies in the DOX-resistant breast cancer cell line (e.g., MCF-7/DOX) are necessary to address its potential to overcome the MDR phenomenon using a similar strategy.

### 2.3. Pharmacokinetics of DOX-Val

**Figure 4:**
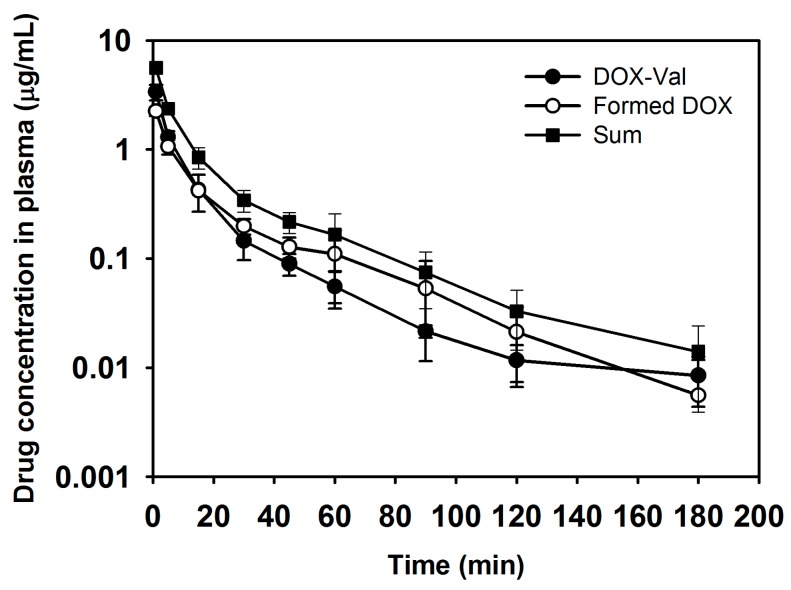
Pharmacokinetic study of DOX-Val (prodrug) after its intravenous administration in rats. DOX-Val (prodrug; ●), formed DOX (metabolite; ○), and the sum of DOX-Val and formed DOX (■) concentrations in plasma according to the time are plotted. Dose of DOX-Val was 4 mg/kg. Data are presented as means ± standard deviation (*n* = 4).

The pharmacokinetics of DOX-Val and free DOX was studied in rats after intravenous bolus administration (Figure 4 and Table 1). DOX-Val was intravenously administered to the rats, and the plasma concentrations of DOX-Val and the formed DOX were quantitatively determined individually. As shown in Table 1, there was no significant difference of AUC values between DOX-Val and the formed DOX. Interestingly, the pharmacokinetic parameters of formed DOX (metabolite) were observed to be similar with those of the prodrug, DOX-Val (Table 1), suggesting the formation rate–limited pharmacokinetics for DOX after DOX-Val administration. Moreover, the sum of AUC values (61.68 ± 8.99 μg·min/mL) for DOX-Val and DOX in this study was comparable to the AUC value (74.26 ± 11.31 μg·min/mL) after the administration of free DOX alone, consistent with the previously reported value (65.18 ± 16.47 μg·min/mL) [27] (Table 1). Other pharmacokinetic parameters of DOX-Val and formed DOX (metabolite) were also changed compared with those values after DOX administration (data not shown). Judging from the AUC values (Table 1), approximately half of the DOX-Val was degraded into DOX in the bloodstream after intravenous administration. Amide bond cleavage may occur by the attack of enzymes (i.e., amidase) in the bloodstream. Although DOX-Val was metabolized to DOX during systemic circulation, the systemic pharmacokinetic studies strongly suggest that remaining DOX-Val in the systemic circulation can exert improved uptake into cancer cells and subsequent enhanced antitumor efficacies, likely via amino acid transporters.

## 3. Materials and Methods

### 3.1. Materials

DOX·HCl was purchased from Boryung Pharmaceutical Co., Ltd. (Seoul, Korea). MCF-7 cells were purchased from the Korean cell line bank (Seoul, Korea). RPMI 1640 (developed by Roswell Park Memorial Institute), penicillin, streptomycin, and fatal bovine serum (FBS) were obtained from Gibco Life Technologies, Inc. (Grand Island, NY, USA). All other reagents were of analytical grade.

### 3.2. Synthesis and Characterization of DOX-Val

#### 3.2.1. *N*-(Valyl-Fmoc)doxorubicin

A solution of Fmoc-Val-OH (10.32 mg, 0.037 mmol) and DOX·HCl (20 mg, 0.032 mmol) was added to dimethyl sulfoxide (DMSO; 1.6 mL). *N*,*N*-Diisopropylethylamine (DIEA; 0.012 mL, 0.068 mmol) was mixed, and the reaction mixture was stirred for 15 min at room temperature. 1-[Bis(dimethylamino)methylene]-1*H*-1,2,3-triazolo[4,5-*b*]pyridinium 3-oxid hexafluorophosphate (HATU) in dimethylformamide (DMF) solution was added. The mixture was stirred for 15 h at room temperature. The residue was dissolved in ethyl acetate and washed with a solution consisting of 10% aqueous citric acid, 10% NaOH solution, and brine. The organic phase was dried over anhydrous MgSO_4_ and evaporated under reduced pressure to dryness. The final residue dissolved in dichloromethane was filtered and dried to afford the crude [22] (28.5 mg, 89% yield) as a red solid which was used without any further purification. Nuclear magnetic resonance (^1^H-NMR) spectra were recorded on a VNMRS-500 (500 MHz (^1^H)) spectrometer using CH_3_OH-*d*_4_ as the solvent, and were reported in ppm relative to CH_3_OH-*d*_4_ (δ 4.78, 3.31) for ^1^H-NMR. Coupling constants (*J*) in ^1^H-NMR are in Hz. ^1^H-NMR (500 MHz, CH_3_OH-*d*_4_): δ 7.52 (m, 2H), 7.34~7.29 (m, 3H), 7.14~7.12 (m, 2H), 7.05~6.98 (m, 2H), 6.95~6.89 (m, 2H), 5.40~5.39 (m, 1H), 4.97 (m, 1H), 4.76~4.75 (m, 2H), 4.38~4.35 (m, 1H), 4.33~4.29 (m, 1H), 4.12~4.08 (m, 1H), 3.88~3.86 (m, 2H), 3.77 (s, 3H), 3.66~3.65 (m, 1H), 3.08~3.05 (m, 2H), 2.13~1.97 (m, 5H), 1.29~1.28 (d, 3H, *J* = 6.2 Hz), 1.25~1.23 (m, 1H), 0.99~0.94 (d, 6H, *J* = 6.5 Hz).

#### 3.2.2. DOX-Val

*N*-(Valyl-Fmoc) doxorubicin (28.2 mg, 0.033 mmol) was added with 50% piperidine in DMF (4 mL). The reaction mixture was stirred at room temperature for 5 min, and the solvent was evaporated under reduced pressure to dry. The crude residue was purified by solidification from hexane-diethyl ether to give DOX-Val (10.1 mg, 48% yield) as a red solid. The purity of DOX-Val was 96.3%, determined by a HPLC system (Shimadzu, Kyoto, Japan), equipped with system controller (CBM-20A; Shimadzu), solvent delivery unit (LC-20A; Shimadzu), auto sampler (SIL-20A; Shimadzu), and photodiode array detector (SPD-M20A; Shimadzu).^1^H-NMR (500 MHz, CH_3_OH-*d*_4_): δ 7.85~7.79 (m, 2H), 7.53~7.51 (m, 1H), 4.79 (m, 2H), 4.33~4.32 (m, 1H), 4.24~4.21 (m, 1H), 4.07~4.03 (m, 5H), 3.64~3.60 (m, 1H), 3.16~3.14 (m, 2H), 2.09~1.80 (m, 5H), 1.11~1.10 (m, 1H), 1.07~0.99 (d, 3H, *J* = 6.7 Hz), 0.94~ 0.92 (d, 6H, *J* = 6.6 Hz).

### 3.3. Cellular Uptake Efficiency Study in MCF-7 Cells

#### 3.3.1. Flow Cytometry

For comparing the cellular uptake efficiency of DOX-Val (prodrug), DOX base (DOX) was prepared by the reported method [27]. Based on the previous observation that the presence of high concentration of glutamine could inhibit the cellular uptake of amino acid-added prodrug [20], MCF-7 cells were cultured with RPMI 1640 medium (no glutamine) containing 10% (*v*/*v*) FBS, 1% (*v*/*v*) penicillin (100 U/mL), and streptomycin (0.1 mg/mL), and they were maintained at 37 °C in a humidified 5% CO_2_ atmosphere. For flow cytometry study, MCF-7 cells were seeded onto six-well plates at a density of 6.0 × 10^5^ cells per well. After incubation for 24 h at 37 °C, the cell culture media were removed, and DOX or DOX-Val solution (10 μM) was added and incubated for 1 h or 3 h. The drug solution was eliminated, and the cells were washed with phosphate buffered saline (PBS, pH 7.4) for at least three times. The cells were detached and collected by centrifugation. Those cells were then resuspended with PBS containing FBS (2%, *v*/*v*). The cellular uptake of DOX and DOX-Val was quantitatively measured by a FACSCalibur fluorescence-activated cell sorter (FACS™) equipped with CELLQuest software (Becton Dickinson Biosciences, San Jose, CA, USA).

#### 3.3.2. CLSM

Cellular distribution of drugs was observed by CLSM imaging. MCF-7 cells were cultured as described above. MCF-7 cells were seeded on culture slides (BD Falcon, Bedford, MA, USA) at a density of 1.0 × 10^5^ cells per well (surface area of 1.7 cm^2^ per well, four-chamber slides) and incubated for 24 h at 37 °C. DOX or DOX-Val (10 μM) was added and incubated for 1 h or 3 h. Then, the cells were washed with PBS (pH 7.4) three times and fixed in a formaldehyde solution (4%, *v*/*v*) for 10 min. After drying under an air stream, VECTASHIELD mounting medium containing 4′,6-diamidino-2-phenylindole (DAPI; H-1200, Vector laboratories, Inc., Burlingame, CA, USA) was treated to the cells on the culture slides. The cells were then observed using CLSM (LSM 710, Carl-Zeiss, Thornwood, NY, USA).

### 3.4. Pharmacokinetic Study

The pharmacokinetics of DOX-Val was evaluated in male Sprague-Dawley (SD) rats (250 ± 5 g of body weight; Orient Bio, Sungnam, Korea). The rats were reared in a light-controlled room with 22 ± 2 °C temperature and 55% ± 5% relative humidity (Animal Center for Pharmaceutical Research, College of Pharmacy, Seoul National University, Seoul, Korea). The experimental protocols of animal studies were approved by the Animal Care and Use Committee of the College of Pharmacy, Seoul National University (No. SNU-160311-3).

For injection of the drug solution and blood sampling, the left femoral vein and artery were cannulated with Intramedic™ polyethylene tube (PE-50; Becton Dickinson Diagnostics, Sparks, MD, USA) under anesthesia with Zoletil (Virbac, Carros, France) with 50 mg/kg dose (intramuscular injection). The DOX-Val and free DOX solution—with a dose of 4 mg/kg—were injected into the femoral vein. Aliquot of blood (~400 μL) was collected from the femoral artery (at 1, 5, 15, 30, 45, 60, 90, 120, and 180 min) and the equivalent volume of normal saline (containing 25 U/mL heparin) was injected at each time point. The collected blood samples were centrifuged at 13,200 rpm for 3 min at 4 °C, and the aliquots (150 μL) of the supernatant were stored at −70 °C prior to its quantitative analysis by the HPLC system. For determining the concentrations of DOX-Val and DOX in the plasma samples, the aliquots of plasma samples (150 μL) were mixed with acetonitrile (550 μL), including daunorubicin (500 ng/mL concentration) as an internal standard (IS) for 10 min, and the mixture was centrifuged at 13,200 rpm for 5 min. The supernatant of centrifuged samples was collected, and the solvent was eliminated by heating at 70 °C under a nitrogen gas stream. Aliquot of mobile phase (60 μL) for HPLC analysis was added to the sample tube and mixed for 5 min. After centrifugation, the aliquot of supernatant (20 μL) was injected into the HPLC system. The concentrations of DOX-Val and DOX in the plasma were quantitatively analyzed using the HPLC system equipped with a pump (PU-2089 Plus; Jasco, Tokyo, Japan), an automatic injector (AS-2050 Plus; Jasco), and a fluorescence detector (FP-2020; Jasco). The mobile phase was consisted of 10 mM KH_2_PO_4_ (pH 2.5 adjusted with phosphoric acid) and 0.1% trimethylamine (TEA) solution in distilled water (DW) (67:33, *v*/*v*), and a reverse phase C18 column (Gemini, 250 mm × 4.6 mm, 5 μm; Phenomenex, Torrance, CA, USA) was used. The flow rate of mobile phase was set at 1 mL/min, and the elution was monitored at 480 nm (excitation) and 560 nm (emission) wavelengths by fluorescence detector. The retention times of DOX, DOX-Val, and IS were 7.9, 9.4, and 17.9 min, respectively. The linearity in the standard samples was established in the range of 10–5000 ng/mL drug concentration in this study. The accuracy and precision values were within acceptable ranges. The pharmacokinetic parameters of DOX-Val, formed DOX (metabolite), sum, and free DOX—total area under the drug concentration–time curve from time zero to time infinity (AUC), terminal half-life (t_1/2_), time-averaged total body clearance (CL), apparent volume of distribution at steady state (V_ss_), and mean residence time (MRT)—were calculated based on non-compartmental analysis by WinNonlin (Version 3.1; Pharsight, Mountain View, CA, USA).

### 3.5. Statistical Analysis

All experiments were performed at least three times, and the data are represented as the means ± standard deviation. A two-tailed Student’s *t*-test was performed to determine significant differences (*p* < 0.05) between the groups.

## 4. Conclusions

DOX-Val was successfully synthesized by amide bond formation between DOX and the Val residue. DOX-Val was efficiently internalized into MCF-7 cells, which express amino acid transporters, compared with DOX. The DOX-Val, as well as DOX, was mainly distributed in the nucleus of the cells, according to the CLSM imaging data. Approximately half of the DOX-Val, based on the AUC values of the DOX-Val and the formed DOX (metabolite), seemed to be metabolized to DOX during systemic circulation. Importantly, the systemic pharmacokinetic studies strongly suggest that the amino acid transporter–targeted amide prodrug (DOX-Val) is metabolically stable in vivo, although the metabolite—DOX—is formed by the metabolism. Synthesized DOX-Val was efficiently internalized into the cancer cells, and subsequent improved antitumor efficacies should be expected.

## Figures and Tables

**Table 1:** Pharmacokinetic parameters of DOX-Val (prodrug), formed DOX (metabolite), and sum in rats after intravenous bolus administration of DOX-Val (*n* = 4).

Pharmacokinetic Parameter	DOX-Val		
	DOX-Val (Prodrug)	Formed DOX (Metabolite)	Sum
AUC (μg∙min/mL)	29.80 ± 4.11	28.58 ± 5.38	61.68 ± 8.99
Terminal t_1/2_ (min)	49.80 ± 7.83	25.59 ± 4.41	33.66 ± 5.43
CL (mL/min/kg)	136.19 ± 19.01	143.39 ± 24.25	65.83 ± 9.10
V_ss_ (mL/kg)	3010.60 ± 619.85	3617.77 ± 625.60	1411.31 ± 245.55
MRT (min)	22.36 ± 5.06	26.35 ± 8.96	22.07 ± 6.59

Administration dose of DOX was 4 mg/kg. Data present as mean ± standard deviation. AUC, total area under the drug concentration–time curve from time zero to time infinity; t_1/2_, terminal half-life; CL, time-averaged total body clearance; V_ss_, apparent volume of distribution at steady state; MRT, mean residence time.
